# Higher hydroxyurea adherence among young adults with sickle cell disease compared to children and adolescents

**DOI:** 10.1080/07853890.2022.2044509

**Published:** 2022-03-02

**Authors:** Paavani S. Reddy, Stephanie W. Cai, Leonardo Barrera, Kathryn King, Sherif M. Badawy

**Affiliations:** a Department of Medical Education, Northwestern University Feinberg School of Medicine, Chicago, IL, USA; b Department of Obstetrics & Gynecology, Northwestern University Feinberg School of Medicine, Chicago, IL, USA; c Mary Ann & J. Milburn Smith Child Health Research, Outreach, and Advocacy Center, Ann & Robert H. Lurie Children’s Hospital of Chicago, Chicago, IL, USA; d Division of Hematology, Oncology, and Stem Cell Transplant, Ann & Robert H. Lurie Children’s Hospital of Chicago, Chicago, IL, USA; e Department of Pediatrics, Northwestern University Feinberg School of Medicine, Chicago, IL, USA

**Keywords:** Sickle cell, adherence, compliance, hydroxyurea, hydroxycarbamide, chronic pain, health care utilization, AYA, adolescents and young adults, haemoglobinopathy

## Abstract

**Background:**

Sickle cell disease (SCD) results in severe complications, such as anaemia and pain episodes. Hydroxyurea (HU) is efficacious in SCD, yet adherence remains low.

**Objective:**

To assess the relationship of HU adherence to health care utilization and patients’ characteristics.

**Methods:**

This is a 5-year retrospective chart review. Patients’ demographics and medical history were collected from the electronic medical record (EMR). HU adherence was evaluated using foetal haemoglobin “HbF%”, mean corpuscular volume “MCV”, and absolute neutrophil count “ANC”. Age groups included children (<12 years), adolescents (12–17 years), and young adults (≥18 years).

**Results:**

A total of 113 SCD patients on HU were included (median age 14 years, IQR 10–20; 50% female; 88% HbSS). Young adults had significantly higher HU adherence compared to adolescents and children, including higher median HbF% (24.2 *vs*. 12.4 *vs*. 8.6, *p* = .003), MCV (fl) (106.4 *vs*. 96.2 *vs*. 95.4, *p* = .01) and lower ANC (10^3^/ml) (3.25 *vs*. 4.9 *vs*. 4.2, *p* = .01), respectively. Patients with chronic pain had lower HU adherence (HbF% 15.3 *vs*. 10.7, *p* = .04; ANC 3.6 *vs*. 6.3, *p* = .002; MCV 102.3 *vs*. 93.1, *p* = .1). Patients with higher HbF or MCV and lower ANC had significantly less frequent emergency room visits (r_s_=–0.26, *p* = .01; r_s_=–0.23, *p* = .01; r_s_=0.24, *p* = .01) and hospitalizations (r_s_=–0.27, *p* = .01; r_s_=–0.31, *p* = .01; r_s_=0.21, *p* = .02) as well as shorter length of stays (r_s_=–0.27, *p* = .0045; r_s_=–.34, *p* = 0.004; r_s_=0.23, *p* = .02), respectively. Similar trends in HU adherence and health care utilization were seen in subgroup analysis of only HbSS patients. There was no significant association of HU adherence to patients’ sex, socio-economic status, distance from hospital, and HU duration.

**Conclusions:**

Young adults with SCD had significantly higher HU adherence compared to children and adolescents. Patients with lower HU adherence and/or chronic pain had increased health care utilization. Future studies examining barriers to adherence and evaluating interventions to optimize HU adherence in SCD are warranted.

**KEY MESSAGES**
1. Young adults with SCD had significantly higher HU adherence, as reflected in their laboratory markers, compared to children and adolescents.
2. Patients with higher HU adherence and/or those without chronic pain had lower or less frequent health care utilization.
3. No significant association of HU adherence to patients’ sex, socio-economic status and distance from hospital.

## Introduction

Sickle cell disease (SCD) is an inherited haemoglobin disorder. SCD is the most common genetic disorder in the United States, especially among individuals of African descent affecting about 1 in 500 African American live births [1–3]. Symptoms of SCD typically begin in early childhood leading to serious complications, including chronic anaemia, vaso-occlusive pain episodes, acute chest syndrome (ACS), stroke, and other end-organ damage [4]. These complications affect patient-reported outcomes (PROs) among individuals with SCD leading to impaired health-related quality of life (HRQOL), in particular physical and mental well-being [5–10]. Earlier studies reported that patients with SCD had higher rates of hospitalizations and emergency room (ER) visits than the general population, particularly readmissions [10,11]. While significant improvements have been made in SCD care, projected life expectancy remains at low 54 years for patients with SCD compared to 76 years for the general population [12].

A cornerstone of SCD care is hydroxyurea (HU), a medication that increases foetal haemoglobin (HbF%), which has protective effects in SCD. While HU was initially only approved for adult populations, FDA approval was officially extended in December 2017 to include paediatric populations age 2 years or older. HU is an efficacious and cost-effective drug in SCD and has been found to reduce disease-related complications and number of hospitalizations with benefits for HRQOL and survival [13–24]. Metformin, a medication commonly used in the treatment of diabetes mellitus, was also found to have some potential clinical benefits in SCD, but the evidence is limited [25]. More recently, the FDA has approved other therapies for SCD, including voxelotor, L-Glutamine, and crizanlizumab [26]. Nevertheless, these new medications are expensive [27], and HU remains the most commonly used medication as a first-line disease-modifying therapy in SCD.

The current literature estimates that HU adherence is suboptimal, falling well below 50% in paediatric, adolescents, and young adults populations [28,29]. Adherence is impacted by a variety of factors or barriers, including fear of side-effects, difficulty communicating with the medical team, misconceptions about HU, and limited access to medications [28–35]. Previously published studies suggested possible predictors of increased health care utilization, such as co-morbidities and living in a remote area with increased distance from the hospital [11,34–37]. Moreover, in SCD, fewer studies have examined the relationship between HU adherence and health care utilization across different age groups, namely children, adolescents, and young adults. Earlier work suggested that adolescents’ adherence may decrease as they transition to young adulthood managing their medications more independently [38], yet a clear relationship between age and adherence has not been established.

The objectives of this study were to: (1) identify potential demographic and clinical correlates of HU adherence among patients with SCD, such as age, socio-economic status, distance from the hospital, duration or formulation of HU, and chronic pain status, and (2) evaluate the relationship between HU adherence and health care utilization, including the number of ER visits and hospitalizations as well as length of hospital stay per year. We hypothesized that some patient characteristics would be associated with higher or lower HU adherence, in particular age. We also hypothesized that higher HU adherence at different time points would be associated with lower health care utilization.

## Materials and methods

### Data source

This is a single-center 5-year retrospective chart review of SCD patients on HU. Clinical and demographic data was extracted from the electronic medical record (EMR) for all patients receiving HU at the comprehensive paediatric and adult sickle cell clinic at Ann & Robert H. Lurie Children’s Hospital of Chicago between June 2012 and June 2017. The study was approved by the Institutional Review Board of Ann & Robert H. Lurie Children’s Hospital of Chicago (IRB 2017-1241).

### Inclusion/exclusion criteria

Patients of any age were included if they had a confirmed diagnosis of SCD, have been on HU for a minimum of 2 years, and were seen at our institution as an outpatient, inpatient or an ER visit during the study time frame. SCD diagnoses were considered confirmed *via* haemoglobin electrophoresis results in the patients’ records. Patients with a diagnosis other than SCD or not on HU were excluded.

### Demographics

Multiple demographic variables were extracted from patients’ EMR, including age at the time of data extraction, sex (male or female), insurance status (insured, uninsured, or unknown), and their geographic living distance from the hospital. Age groups were categorized as children (<12 years), adolescents (12–17 years), and young adults (≥18 years). Utilizing the mean income based on the patients’ geographic zip code, patients were categorized into four groups based on the Illinois statewide median income: Group 1: < 60% of statewide median (<$38,145), Group 2: ≥60% to < 100% of statewide median (≥$38,145 to < $63,575), Group 3: ≥100% to <140% of statewide median (≥$63,575 to <$89,005), Group 4: ≥140% of statewide median (≥$89,005) [39]. Average income per neighbourhood was determined using the 2018 US census data for Illinois [40].

### Treatment history and health care utilization

Data were collected for several variables related to SCD treatment with HU, including dose, formulation (liquid or tablet form), indication, and the approximate duration of HU treatment at the time of data extraction. Maximum tolerated dose was 35 mg/kg/d as supported by Ware 2010 and Heeney 2016 [41,42]. Chronic pain data were recorded from EMR to determine whether or not patients had chronic pain specifically documented in their chart or had a prescription for daily opioid use. Data for surgical procedures to treat SCD-related complications, such as splenectomy and cholecystectomy, were recorded. Additionally, inpatient hospitalizations, duration or length of stay (LOS), and ER visits were also extracted and reported as a rate of events per year given the variability in follow-up period across study cohort.

### Laboratory markers of HU adherence

Laboratory values for common markers used in the treatment adherence and monitoring of SCD were extracted and recorded for the analysis in this study. These adherence markers included foetal haemoglobin (HbF%), mean corpuscular volume (MCV), and absolute neutrophil count (ANC). Laboratory markers from the most recent visit of the study period were obtained. The median over the last 1 year of the study period (June 2016 – June 2017), and median over the last 2 years (June 2015 – June 2017) for each marker was also calculated.

### Statistical analysis

Descriptive statistics were performed on the key demographic variables from the patients’ data and reported as median (interquartile range or IQR), frequencies and percentages. Laboratory markers of adherence, including HbF%, MVC, and ANC (most recent, last 1 year, and last 2 years), were analysed, in relation to different demographics (e.g. age group, sex, and socio-economic status), clinical variables (e.g. chronic pain status, HU duration, and formulation) and health care utilization (e.g. ER visits, hospitalizations, and LOS). Differences for categorical variables were examined by using the Chi-square test. The Kruskal–Wallis test was used to evaluate for differences in a continuous dependent variable by a categorical independent variable (with two or more groups), and Spearman’s rank correlation was used for continuous variables. Sub-group analyses were performed in patients with HbSS genotype.

## Results

### Participants’ characteristics

Our study included 113 individuals who were 50.4% female, with a median age of 14 years old (IQR 10–20) (Table 1). More than half of patients had private insurance (56.6%) and lived a median distance of 15.2 miles (IQR 9.5–30.9) from the hospital. Patients were prescribed HU mainly for recurrent pain episodes (46.1%) with a median dose of 27.5 mg/kg/dose (IQR 23.25–30.75). The median number of SCD-related ER visits and hospitalizations were 0.27 and 0.33 per year, respectively, with a median LOS of 1 day annually. Markers of HU adherence included a median HbF% of 14.5% (IQR 8.2–28.05), MCV of 99.6 fl (IQR 90.4–112.5), and ANC of 3.84 10^3^/mL (IQR 2.49–5.93).

**Table 1.** Participants’ characteristics.

	All (*N* = 113)	HbSS only (*N* = 99)
Age (years), median (IQR)	14 (10–20)	14 (10–28)
Age group (years), *n* (%)		
Children (<12)	37 (32.7%)	29 (29.3%)
Adolescents (12–17)	26 (23%)	26 (26.3%)
Young adults (≥18)	50 (44.3%)	44 (44.4%)
Female, *n* (%)	57 (50.4%)	52 (52.53%)
Sickle cell genotype, *n* (%)		
HbSS	99 (87.6%)	99 (100%)
HbSC	9 (8%)	–
Other^a^	5 (4.4%)	–
HU indication, *n* (%)		
Recurrent pain	35 (46.1%)	25 (38.5%)
CNS disease^b^	11 (14.5%)	11 (16.92%)
Recurrent acute chest syndrome	5 (6.6%)	13 (20%)
≥1 of above indications	11 (14.5%)	11 (16.92%)
Others^c^	12 (17.2%)	13 (20%)
HU dose (mg/kg/dose), median (IQR)	27.5 (23.3–30.8)	28 (24–32)
HU formulation, *n* (%)		
Liquid	18 (16.2%)	17 (17.5%)
Tablet	93 (83.8%)	80 (82.5%)
HU MTD,^d^ *n* (%)	13 (11.6%)	13 (13.4%)
HU duration (months), median (IQR)	47 (20–60)	47 (20–60)
Chronic pain, *n* (%)	20 (17.7%)	17 (17.2%)
Cholecystectomy, *n* (%)	19 (16.8%)	17 (17.2%)
Splenectomy, *n* (%)	11 (9.7%)	9 (9.1%)
Insurance, *n* (%)		
Private	64 (56.6%)	60 (60.6%)
Public/Medicaid	44 (38.9%)	34 (34.3%)
Combined^e^	4 (3.5%)	4 (4%)
None	1 (0.9%)	1 (1%)
Distance to hospital (miles), median (IQR)	15.2 (9.5–30.9)	14.3 (9.5–30.6)
Laboratory markers (most recent), median (IQR)		
Foetal haemoglobin (%)	14.5 (8.2–28.1)	15.3 (9.1–28.8)
Mean corpuscular volume (fl)	99.6 (90.4–112.5)	102.3 (92.6–112.9)
Absolute neutrophil count (10^3^/ml)	3.8 (2.5–5.9)	3.8 (2.4–6)
SCD emergency room visits on HU (rate/year), median (IQR)	0.27 (0–2.0)	0.2 (0–1.6)
SCD hospitalizations on HU (rate/year), median (IQR)	0.33 (0–1.4)	0.24 (0–1.4)
SCD inpatient LOS on HU (days/year), median (IQR)	1 (0–20.6)	0.67 (0–5.2)

HU: hydroxyurea; LOS: length of stay; MTD: maximum tolerated dose; SCD: sickle cell disease.

^a^Other genotype included HbSβ^+^ (*n* = 2), HbSβ^0^ (*n* = 2), HbS/Alpha-Thalassemia (*n* = 1).

^b^CNS disease defined as stroke or abnormal transcranial Doppler.

^c^Other hydroxyurea indications included poor growth (*n* = 5), anaemia (*n* = 3), transition from chronic transfusion (*n* = 3), hypoxia (*n* = 1), and abnormal imaging (*n* = 1). The remainder were not located in the EMR (*n* = 37).

^d^Maximum tolerated dose was 35 mg/kg/d.

^e^Combined insurance is referring to patients who had both public then private insurance over time, or vice versa.

### HU adherence and patient characteristics

Patients’ age group and chronic pain status were significantly associated with difference in laboratory markers of adherence (Table 2). Young adults with SCD demonstrated a higher level of HU adherence in their most recent median HbF% (24.15 *vs*. 12.4 *vs*. 8.6, *p* = .0003) and most recent median MCV values (106.4 *vs*. 96.2 *vs*. 95.4, *p* = 0.01), compared to adolescents and children, respectively. This finding was also consistent for HbF and MCV values across 1- and 2-year follow-up periods as well. Furthermore, ANC values over the course of 1 year follow up were also significantly lower among young adults (Table 2), suggesting higher HU adherence, compared to children and adolescents (3.25 *vs*. 4.93 *vs*. 4.21, *p* = .01), respectively.

**Table 2.** Laboratory markers of hydroxyurea adherence in relation to patients’ age and chronic pain status.

All participants (*N* = 113)							
	Age groups (years)				Chronic pain		
	<12 (*n* = 37)	12–17 (*n* = 26)	≥18 (*n* = 50)	*p* Value	Yes (*n* = 20)	No (*n* = 93)	*p* Value
HbF, median (IQR)							
Most recent	8.6 (5.1–20.8)	12.4 (9.9–16.3)	24.2 (11.7–30.3)	**.003**	10.7 (4.6-19.5)	15.3 (8.9-29.3)	**.04**
Last year	8.4 (5.1–21.2)	14 (10.5–20.7)	24.4 (17.7–24.1)	**.001**	14.5 (4.9-20.7)	18 (9.8-29.4)	.24
Last 2 years	10.4 (5.1–15.5)	16.7 (12.4–24.4)	21.4 (15.2–26.4)	**.01**	17.4 (8.1-22.5)	16.1 (10.9-25)	.58
MCV, median (IQR)							
Most recent	95.4 (81.6–102.4)	96.2 (90.9–113.1)	106.4 (92.6–116)	**.01**	93.1 (87.9–1003.5)	102.3 (91.4–112.7)	.1
Last year	96.4 (85.1–100.4)	94.6 (92.1–113.4)	106.2 (95.5–114.6)	**.04**	95.7 (89–112.1)	99.1 (92–113.4)	.46
Last 2 years	94.5 (85.7–101.1)	97.5 (92.3–112.5)	109.8 (97.3–113.7)	**.01**	96.6 (92.1–109.8)	100.8 (91.9–112.5)	.29
ANC, median (IQR)							
Most recent	5 (2.9–5.9)	3.7 (2.7–6.1)	3.61 (2.3–5.7)	.5	6.3 (4–7)	3.6 (2.3–5.6)	**.002**
Last year	4.2 (3.4–6.8)	4.9 (3.6–6.6)	3.25 (2.4–4.7)	**.01**	4.2 (3.5–5)	3.77 (3–5.7)	.66
Last 2 years	4.4 (3.1–6.7)	4.8 (3.8–7)	3.9 (2.8–5.4)	.16	4.4 (3.9–6.2)	4.2 (3.1–6)	.6

Participants with HbSS (*N* = 99)							
	Age groups (years)				Chronic pain		
	<12 (*n* = 29)	12–17 (*n* = 26)	≥18 (*n* = 44)	*p* Value	Yes (*n* = 17)	No (*n* = 72)	*p* Value
HbF, median (IQR)							
Most recent	9.25 (6.6–26.5)	12.4 (9.9–16.3)	24.3 (13.6–33.1)	**.01**	12.8 (8.7–22.6)	18.4 (9.1–30.3)	.12
Last year	9.55 (6.45–25.3)	14 (10.5–20.7)	24.4 (17.7–32.7)	**.001**	15.9 (9.0–22.8)	18.8 (10.5–29.4)	.29
Last 2 years	10.8 (5.3–21.6)	16.7 (12.4–24.4)	21.4 (15.2-26.4)	**.03**	17.8 (9.0–24.0)	16.8 (12.4–25)	.74
MCV, median (IQR)							
Most recent	99.3 (92.5–104.9)	96.2 (90.9–113.1)	107.5 (93.6–116.1)	.08	96 (91.7–106.6)	104.1 (93.6–113.1)	.17
Last year	98.9 (94.9–104.5)	94.6 (92.1–113.4)	107 (95.8–114)	.18	95.9 (91.1–113.3)	100 (92.8–114)	.41
Last 2 years	96.7 (91.3–101.2)	97.5 (92.3–112.5)	110.9 (97.3–115.2)	**.02**	96.7 (92.5–110.4)	101.1 (94.8–113.7)	.28
ANC, median (IQR)							
Most recent	5.1 (2.4–6.4)	3.7 (2.7–6.1)	3.5 (2.3–5.7)	.48	6.6 (6.9–3.5)	3.6 (2.3–5.6)	**.003**
Last year	4.2 (3.6–6.8)	4.9 (3.6–6.6)	3.2 (2.4–4.2)	**.002**	4.1 (3.2–5.9)	3.7 (3–5.5)	.65
Last 2 years	4.7 (4.1–7.7)	4.8 (3.8–7)	3.7 (2.7–5.2)	**.04**	4.3 (3.8–6.3)	4.2 (3.1–6.1)	.75

*p* <.05 was statistically significant (highlighted in bold).

ANC: absolute neutrophil count; HbF: foetal haemoglobin; IQR: inter-quartile range; MCV: mean corpuscular volume.

With regard to patients’ chronic pain status, the most recent laboratory value for median HbF% and MCV were found to be higher in individuals without chronic pain (15.3 *vs*. 10.7, *p* = .04; 102.3 *vs*. 93.1, *p* = .1), suggesting an association between higher HU adherence and lower risk of developing chronic pain. Similarly, ANC was found to be significantly lower in individuals without chronic pain, further corroborating that higher HU adherence might have some protective effects among SCD individuals without chronic pain (3.58 *vs*. 6.28, *p* = .002) (Table 2). In addition, these findings were observed in a subgroup analysis of HbSS patients only (Table 2).

### HU adherence and health care utilization

Patient health care utilization was collected across a 5-year study period. Patients with higher HbF, MCV or both, and those with lower ANC values, all of which are associated with greater adherence to HU, had significantly fewer SCD-related ER visits and hospitalizations as well as shorter LOS for their hospitalizations (Table 3). Similar findings were also seen in a subset analysis of HbSS patients only (Table 3).

**Table 3.** Association between laboratory markers of hydroxyurea adherence and healthcare utilization.

All participants (*N* = 113)												
	Emergency room visits (rate/year)				Hospitalizations (rate/year)				LOS hospitalizations (days/year)			
	All (r_s_)	*p* Value	HU (r_s_)	*p* Value	All (r_s_)	*p* Value	HU (r_s_)	*p* Value	All (r_s_)	*p* Value	HU (r_s_)	*p* Value
HbF												
Most recent	−0.18	.07	−0.26	**.01**	−0.21	**.03**	−0.27	**.01**	−0.23	**.02**	−0.27	**.0045**
Last 1 year	−0.27	**.02**	−0.30	**.01**	−0.25	**.03**	−0.30	**.01**	−0.26	**.02**	−0.32	**.004**
Last 2 year	−0.14	.36	−0.01	.32	−0.25	**.04**	−0.28	**.02**	−0.27	**.03**	−0.31	.01
MCV												
Most recent	−0.20	**.04**	−0.23	.01	−0.18	.06	−0.14	.25	−0.22	**.02**	−0.14	.13
Last 1 year	−0.20	.08	−0.15	.17	−0.19	.08	−0.11	.32	−0.24	**.03**	−0.12	.27
Last 2 year	−0.24	.05	−0.18	.07	−0.31	.01	−0.17	.15	−0.34	**.004**	−0.19	.11
ANC												
Most recent	0.23	**.02**	0.24	**.01**	0.22	**.02**	0.21	**.02**	0.22	**.02**	0.23	**.02**
Last 1 year	0.25	**.02**	0.25	**.03**	0.18	.11	0.17	.12	0.14	.20	0.15	.27
Last 2 year	0.14	.24	0.06	.66	0.18	.13	0.15	.22	0.16	.19	0.15	.23

Participants with HbSS (*N* = 99)												
	Emergency room visits (rate/year)				Hospitalizations (rate/year)				LOS hospitalizations (days/year)			
	All (r_s_)	*p* Value	HU (r_s_)	*p* Value	All (r_s_)	*p* Value	HU (r_s_)	*p* Value	All (r_s_)	*p* Value	HU (r_s_)	*p* Value
HbF												
Most recent	−0.15	.14	−0.20	**.045**	−0.18	.09	−0.25	**.015**	−0.18	.08	−0.25	**.01**
Last 1 year	−0.21	.07	−0.21	.07	−0.17	.14	−0.22	.07	−0.17	.2	−0.23	**.05**
Last 2 year	−0.05	.56	−0.02	.87	−0.16	.22	−0.19	.15	−0.17	.2	−0.20	.11
MCV												
Most recent	−0.16	.13	−0.14	.16	−0.16	.13	−0.11	.27	−0.22	**.03**	−0.12	.26
Last 1 year	−0.13	.25	−0.02	.88	−0.12	.29	−0.004	.97	−0.18	.11	−0.01	.93
Last 2 year	−0.24	.06	−0.08	.54	−0.27	**.04**	−0.15	.26	−0.31	**.01**	−0.16	.22
ANC												
Most recent	0.21	**.04**	0.22	**.03**	0.26	.01	0.22	**.03**	0.27	**.01**	0.24	**.02**
Last 1 year	0.24	**.04**	0.23	**.04**	0.19	.11	0.16	.17	0.15	.18	0.16	.19
Last 2 year	0.16	.22	0.07	.60	0.24	.06	0.17	.19	0.22	.09	0.18	.16

*p* < 0.05 was statistically significant (highlighted in bold).

r_s_, Spearman rho correlations; “All” is defined as entire follow up period, including HU therapy; “HU” is defined as being on HU.

ANC: absolute neutrophil count; HbF: foetal haemoglobin; HU: hydroxyurea; LOS: length of stay; MCV: mean corpuscular volume.

### Other correlates of adherence and health care utilization

Males were more likely to have higher HU adherence as demonstrated in their median [IQR] MCV values (105.8 [96–114.6] *vs.* 95.6 [91–107.6], *p* = .02), compared to females, respectively. However, we found no statistically significant relationship of laboratory markers of HU adherence to patients’ SES, insurance status, or distance from the hospital (Supplemental Table 1). Patients’ MCV values positively correlated with duration of HU therapy (r_s_=0.28, *p* = .02), suggesting higher adherence overtime. In addition, patients on HU tablet formulation were more likely to have higher adherence levels compared to patients on liquid, indicated in both their MCV values (102.4 [93.6–114.8] *vs*. 94.5 [89.6–104.9], *p* = .03) and HbF% (18.1 [12.4–25.6] *vs*. 14.6 [5.3–17.7], *p* = .07), respectively (Supplemental Table 2).

There was a statistically significant relationship between chronic pain status and health care utilization as demonstrated by ER visits, hospitalizations, and LOS. Patients with a chronic pain status had more frequent median ER visits per year (2.17 *vs*. 0, *p* = .001) and hospitalizations per year (1.8 *vs*. 0, *p* < .001) as well as longer LOS in a year (9.6 *vs*. 0, *p* < .001), compared to those without chronic pain (Table 4). Lastly, compared to adolescents, we found that young adults with SCD had less frequent median annual ER visits and hospitalizations as well as shorter LOS (0.4 *vs*. 0.2; 0.5 *vs*. 0.2; 1.7 *vs*. 0.64), respectively; however, these were not statistically significant. Similar trends were seen in a sub-group analysis that included only HbSS patients.

**Table 4.** Health care utilization in relation to patients’ age and chronic pain status, and duration of prescription.

All participants (*N* = 113)							
	Age groups (years)				Chronic pain		
	<12	12–17	≥18	*p* Value	Yes	No	*p* Value
Emergency room, median (IQR)							
All	1.1 (0.3–2.2)	1.1 (0–2.0)	0.24 (0–2.0)	.32	1.9 (1.1–5.0)	0.6 (0–1.8)	**<.001**
On HU	1 (0-2.2)	0.40 (0-2.0)	0.20 (0-1.5)	.58	2.17 (1–4.25)	0 (0–1.4)	**.001**
Hospitalization, median *(*IQR)							
All							
Stays a year	0.6 (0–1.4)	0.74 (0.4–1.8)	0.4 (0–1.6)	.57	1.6 (0.5–4.2)	0.4 (0–1.2)	**.001**
Length of stay	2.0 (0–7.6)	3.95 (1.2–9.4)	1.8 (0–7.0)	.44	9.6 (3.2–24)	1.8 (0–7.0)	**<.001**
On HU							
Stays a year	0 (0–1.4)	0.5 (0–1.6)	0.2 (0–1.4)	.45	1.8 (0.43–3.5)	0 (0–1.0)	**<.001**
Length of stay	0 (0–6.4)	1.7 (0–8.6)	0.64 (0–7.8)	.53	9.64 (2.87–3.3)	0 (0–3.0)	**<.001**

Participants with HbSS (*N* = 99)							
	Age groups (years)				Chronic pain		
	<12	12–17	≥18	*p* Value	Yes	No	*p* Value
Emergency room, median *(*IQR)							
All	0.9 (0–2.0)	1.1 (0–2.0)	0.24 (0–2.0)	.56	2.0 (1.5–4.0)	0.4 (0–1.8)	**<.001**
On HU	0 (0–2.2)	0.40 (0–2.0)	0.20 (0–1.4)	.66	2.33 (1.4–4.0)	0 (0–1.0)	**<.001**
Hospitalization, median *(*IQR)							
All							
Stays a year	0.4 (0–1.2)	0.74 (0.4–1.8)	0.4 (0–1.6)	.37	2.0 (0.8–4.4)	0.4 (0–1.2)	**<.001**
Length of stay	1.8 (0–6.4)	3.95 (1.2–9.4)	1.6 (0–6.9)	.25	15 (3.4–25.6)	1.4 (0–5.2)	**<.001**
On HU							
Stays a year	0 (0–1)	0.5 (0–1.6)	0.2 (0–1.4)	.26	2.0 (0.8–4.0)	0 (0–0.74)	**<.001**
Length of stay	0 (0–3.6)	1.7 (0–8.7)	0.67 (0–6.4)	.28	15 (3.4–25.6)	0 (0–2.44)	**<.001**

*p* <.05 was statistically significant (highlighted in bold).

HU: hydroxyurea; IQR: inter-quartile range.

“All” is defined as entire follow up period, including being HU therapy; “On HU” is defined as the period being on HU.

## Discussion

This study contributes to the growing body of literature on HU adherence and health care utilization in SCD. We found that young adults had significantly higher adherence to HU, as reflected in their laboratory markers, compared to children and adolescents. Patients with higher HbF% and/or MCV as well as lower ANC had significantly less frequent annual ER visits and hospitalizations with shorter LOS. Patients with chronic pain status had significantly lower HU adherence as well as increased health care utilization, compared to those without chronic pain. Male sex, longer duration of HU therapy, and tablet formulation were associated with higher HU adherence overtime.

Our data also showed that higher HU adherence correlated with decreased health care utilization. This finding is consistent with other published studies that have examined the relationship between HU adherence and health care utilization [21,43–46]. Health care utilization is often a reflection of disease severity, specifically the burden of SCD complications, and has many implications for long-term health outcomes, morbidity, and early mortality [47]. HU has been found to be both efficacious and of high clinical value in terms of reducing morbidity and mortality and to lower health care utilization in patients with SCD [48,49]. In the HUSTLE trial, published in 2017, higher HU adherence, as indicated by HbF ≥20%, was associated with decreased SCD-related complications and less frequent hospitalizations [44]. Similarly, a single-center study of 37 paediatric patients in the United Kingdom showed that higher HU adherence was significantly associated with less frequent SCD-related hospitalizations [45]. Furthermore, we found that chronic pain was significantly associated with lower HU adherence and increased health care utilization, which is consistent with the published literature [23]. The association of chronic pain with both adherence and health care utilization aligns with our hypothesis that factors associated with adherence would also be associated with health care utilization.

In our study, we sought to examine the relationship of different patients’ characteristics in relation to HU adherence and health care utilization. In particular, we found that young adults had statistically significant higher HU adherence, as demonstrated in laboratory markers (i.e. HbF%, MCV, and ANC), compared to children and adolescents. The relationship between age and adherence has not been established in the published literature [38]. Moreover, we anticipated that, if there was a relationship between age and adherence, younger populations would have greater adherence due to parental motivation and co-management of their care. One study in North Carolina examined the records of 2,790 patients with SCD and found that co-management (involvement of both a primary care provider and haematologist in SCD management) was associated with higher adherence [50]. Co-management was also higher amongst the younger age groups (<18 years) compared to the adult sub-groups [50].

Recall barriers, or forgetfulness, is the most common reason for low adherence or non-adherence in the general population and in patients with SCD [32,51–53]. In addition, having multiple parties involved in remembering or managing a medication regimen could be an asset or hinderance to adherence in younger patients. Moreover, there are many more HU adherence challenges among young adults, including a transition to independently managing medications and their SCD as well as other psychosocial stressors during the transition from adolescence to adulthood [52]. Conversely, there are a few factors that may support our findings of young adults in our cohort having increased HU adherence. Adult populations maintain greater autonomy and independence in their medication regimen, and thus may be more motivated to adhere to their regimen. Our findings were also notable as the “adolescent” cohort had greater adherence than the paediatric cohort, but lower than the adult cohort, as this is the age group where patients transition in their autonomy, independence, and involvement of parents in their care. This is especially significant and interesting, as some studies have suggested that adolescents may have unique challenges with adherence, particularly related to negative beliefs and concerns about HU [32,33,52,54–56]. A study of self-reported barriers to adherence in patients’ age 13–24 years old found that patients craved greater independence in managing their medication, while also fearing bullying and stigmatization from being labelled as a patient when taking their medications [52]. Haywood et al. [55] have reported that patients’ concerns about HU may also relate to the following: no perceived benefit, lack of knowledge, and side-effects (e.g. safety, reproductive effects, and carcinogenicity). It is also worth noting that physicians, patients, and family members may have different outlooks towards a patient’s disease severity. In a 2005 study by Connelly et al. [57], parents and physicians of patients with SCD reported more SCD-related symptoms and increased disease severity than the patients themselves. As a result of these factors, although medication non-adherence has generally been high among the paediatric chronic health patients, estimates of adolescent non-adherence are often even higher [52,53].

There are factors that could support increasing adherence with age, including throughout adolescence. The transition to adulthood allows for even greater independence, control, and self-care which can be motivating to patients. Patients may appreciate the benefits and necessity of a medication in managing their symptoms based on their own experiences to a greater degree than a parent or caregiver might. This has been observed in patients with cystic fibrosis, where older adolescents and young adults (AYA) have been found to perceive a higher necessity of enzyme supplement, vitamins, and chest physiotherapy [57,58]. Previous studies have found that individuals with SCD across all ages have high health care utilization and more frequent hospitalizations and ER visits; this is especially true for AYA have who have been found to have longer hospital length of stays (LOSs) as well [11,36,37,59–61]. Another consideration would be that young adults learned the skills of adherence overtime. However, given the variability in adherence and duration of medication use within each age group of our study, this is less likely to explain the trends observed in this data.

While patient age proved to be a significant finding in relation to HU adherence, it is worthwhile to note that many demographic qualities, such as SES, patient insurance status and distance from the hospital were *not* significantly associated with either adherence or health care utilization. The lack of association with insurance status may be partially due to the paediatric population in this study, where almost all of the participants were insured, many by Medicaid (38.9%). In future studies, it may be worthwhile to examine the relationship between SES and distance from the hospital in a cohort that includes both insured and uninsured individuals. Further, patients had an average of 15.2 miles from the hospital, and thus may not have the best indicator for the effect of large distance of travel time for patients, which warrant further evaluation in future studies in larger metropolitan areas or health systems.

### Strengths and limitations

A major strength of the study is its focus on children, adolescents and young adults with SCD, which are vulnerable populations known to have more frequent disease-related complications [62]. Moreover, the time course of our study (5 years) including analyses of laboratory markers of adherence at various timepoints, namely most recent, 1 year and 2 years, enabled us to examine both short- and long-term adherence in this population with relatively consistent results. However, our study has few limitations as well. This study was conducted at a single academic institution, which relatively limited our cohort sample size and generalizability of our findings, given study setting. In addition, our data on health care utilization in particular relied on using the number of SCD events (i.e. ER visits, hospitalizations, and LOS) in our institution’s EMR. It is possible that we may have missed events at other institutions that were not reflected or documented in our medical records. Finally, another potential inherent limitation in our study was its design as a retrospective chart review. Our adherence was measured primarily through laboratory values known to be strong markers or surrogate of adherence. Other measures of adherence, such as subjective self-report surveys, electronic pill bottles, pill count, and/or patient medication logs could have provided more insight into patients’ adherence behaviour as well as their different perceived barriers to adherence.

## Conclusions

Adherence to HU remains a challenge in the SCD populations. Young adults had significantly higher HU adherence compared to children and adolescents with SCD. Research in this domain is crucial precisely due to the vulnerability of these populations, and as SCD is a chronic illness, identifying factors that impact adherence early on can have a lifelong impact on patients. Our findings enhance our understanding of health care utilization in patients with SCD and underscores the importance of adherence in the outcomes of SCD patients. Further studies are needed to better understand the relationship of HU adherence dynamics to age, transition of care, SES, distance from the hospital, insurance status, and other demographic factors. Specifically, future longitudinal studies could examine the impact of patient autonomy, parental involvement, health literacy, and social support on HU adherence overtime, which would be crucial to identify barriers and facilitators of adherence. Moreover, future studies are needed to investigate the potential role of behavioural interventions, especially digital ones, to improve HU adherence in SCD. Several ongoing studies right now are looking at interventions to improve adherence in this population, such as the addition of a community health worker, and use of a mobile health app [63–65]. These studies emphasize the importance of the parent–youth dyad in their interventions, which could address some of the challenges with adherence experienced by the younger age groups in this study [63]. Optimizing adherence to HU and other disease modifying therapies is key to improve health outcomes and HRQOL as well as decrease risk of complications and early mortality in this vulnerable population of children, adolescents, and young adults with SCD.

## Supplementary Material

Supplemental Material

Supplemental Material

## Data Availability

The authors confirm that the data supporting the findings of this study are available within the article [and/or] its supplementary materials. Data can be shared upon request by contacting the corresponding author.
