# Supplementary material for: Higher hydroxyurea adherence among young adults with sickle cell disease compared to children and adolescents
Source: Ann Med. 2022 Mar 2;54(1):683–93. doi: 10.1080/07853890.2022.2044509 (PMC8896206; doi:10.1080/07853890.2022.2044509)
Supplement: Supplemental Material [file IANN_A_2044509_SM5767.docx]

**Supplemental 2. Laboratory markers of hydroxyurea adherence in relation to patients’ hydroxyurea formulation and duration**

| **All Participants (N=113)** | | | | | |
| --- | --- | --- | --- | --- | --- |
|  | **Formation** | | | **Duration** | |
|  | **Liquid** | **Tablet** | ***P* value** | **Correlation (r_s_)** | ***P* value** |
| **HbF** | Median, IQR | | | Correlation | |
| Most recent | 16.1 (9.2-34.0) | 13 (8.0-27.2) | 0.29 | -0.001 | 0.98 |
| Last year | 17.9 (7.2-31.3) | 17.9 (8.7-26.2) | 0.64 | -0.01 | 0.92 |
| Last 2 years | 14.6 (5.3-17.7) | 17.6 (10.8-25.6) | 0.16 | -0.1 | 0.44 |
| **MCV** | Median, IQR | | | Correlation | |
| Most recent | 94.1 (88.8-104.9) | 100.6 (90.9-113.1) | 0.09 | 0.23 | **0.01** |
| Last year | 98.1 (92.1-108.1) | 98.6 (89.7-113.8) | 0.67 | 0.21 | **0.05** |
| Last 2 years | 96.6 (92.9-102.2) | 100.2 (91.4-112.9) | 0.22 | 0.28 | **0.02** |
| **ANC** | Median, IQR | | | Correlation | |
| Most recent | 3.71 (2.35-5.92) | 3.85 (2.53-5.93) | 0.79 | 0.03 | 0.78 |
| Last year | 3.67 (3.14-5.18) | 4.17 (2.96-5.74) | 0.67 | -0.05 | 9.67 |
| Last 2 years | 4.77 (4.12-7.59) | 4.23 (3.09-5.71) | 0.22 | -0.16 | 0.18 |
|  | | | | | |
| **Participants with HbSS (N=99)** | | | | | |
|  | **Formation** | | | **Duration** | |
|  | **Liquid** | **Tablet** | ***P* value** | **Correlation (r_s_)** | ***P* value** |
| **HbF** | Median, IQR | | | Correlation | |
| Most recent | 16.1 (9.2-34.0) | 14.7 (9.0-28.5) | 0.54 | -0.07 | 0.48 |
| Last year | 17.9 (7.2-31.3) | 10.8 (10.5-26.2) | 0.86 | -0.06 | 0.6 |
| Last 2 years | 14.6 (5.3-17.7) | 18.1 (12.4-25.6) | 0.07 | 0.06 | 0.67 |
| **MCV** | Median, IQR | | | Correlation | |
| Most recent | 94.5 (89.6-104.9) | 102.4 (93.6-114.8) | **0.03** | 0.03 | 0.74 |
| Last year | 94.5 (89.6-104.9) | 102.4 (92.4-114.0) | 0.70 | -0.02 | 0.88 |
| Last 2 years | 99.1 (94.6-108.1) | 100.0 (92.7-114.4) | 0.15 | -0.19 | 0.13 |
| **ANC** | Median, IQR | | | Correlation | |
| Most recent | 3.9 (2.35-5.92) | 3.77 (2.51-5.99) | 0.93 | 0.17 | 0.09 |
| Last year | 3.60 (3.14-5.18) | 3.86 (2.99-5.33) | 0.81 | 0.14 | 0.23 |
| Last 2 years | 4.77 (4.12-7.59) | 4.23 (3.09-6.05) | 0.25 | 0.25 | 0.048 |

P value <0.05 was statistically significant (highlighted in bold)

r_s_, Spearman rho correlations

ANC: absolute neutrophil count; HbF: fetal hemoglobin; MCV: mean corpuscular volume
