# Supplementary material for: Higher hydroxyurea adherence among young adults with sickle cell disease compared to children and adolescents
Source: Ann Med. 2022 Mar 2;54(1):683–93. doi: 10.1080/07853890.2022.2044509 (PMC8896206; doi:10.1080/07853890.2022.2044509)
Supplement: Supplemental Material [file IANN_A_2044509_SM5764.docx]

**Supplemental Table 1. Laboratory markers of hydroxyurea adherence in relation to patients’ sex and socio-economic status**

| **All Participants (N=113)** | | | | | | | | |
| --- | --- | --- | --- | --- | --- | --- | --- | --- |
|  | **Sex** | | | **Socio-economic status*** (Statewide median income=$63575) | | | | |
|  | **Male** | **Female** | ***P* value** | **Group 1** | **Group 2** | **Group 3** | **Group 4** | ***P* value** |
| **HbF**, median (IQR) | | | | | | | | |
| Most recent | 14.5 (8.8 - 26.5) | 14.4 (6.7 -29.2) | 0.9 | 15.5 (6.6 - 27.6) | 13 (8.7 - 29.2) | 16.3 (6.1 - 26.1) | 14.4 (10.2 - 26.6) | 0.99 |
| Last year | 17.5 (10.6 - 28.3) | 18.1 (8.1 - 29.4) | 0.95 | 18.8 (10.3 - 26) | 16.7 (8.2 - 24.9) | 28.4 (7.2 - 34.2) | 17.5 (10.9 - 24.5) | 0.52 |
| Last 2 years | 15.8 (9.9 - 24.7) | 18 (10.8 - 25.6) | 0.44 | 15.2 (2.1 - 5.6) | 16.4 (9.9 - 24) | 25 (7.9 - 37.3) | 16.5 (12.4 - 19.2) | 0.46 |
| **MCV**, median (IQR) | | | | | | | | |
| Most recent | 98.4 (91 -108.6) | 100.7 (89.7 -113) | 0.51 | 99.6 (92.6 -108) | 99 (86.4 - 112.6) | 104 (96 - 112.5) | 101.6 (91.6-120) | 0.7 |
| Last year | 100 (92.5 - 114.5) | 95.3 (90 - 107) | 0.11 | 98.5 (95 - 108.8) | 98.6 (89 - 113.5) | 99.2 (94 - 105.8) | 101 (94.6-119.8) | 0.73 |
| Last 2 years | 100.9 (90 - 110.9) | 97.2 (92.1- 112.9) | 0.98 | 98.6 (94.8 -104.9) | 96.7 (89.6 - 111) | 101.6 (98 - 116) | 100.9 (93.7-112.9) | 0.39 |
| **ANC**, median (IQR) | | | | | | | | |
| Most recent | 3.8 (2.3 - 6.4) | 3.10 (2.72 - 5.75) | 0.94 | 4.5 (2.9 - 5.9) | 3.8 (2.4 - 6.1) | 3.7 (3.1 - 5.7) | 3.6 (2.1 - 6.8) | 0.92 |
| Last year | 4 (3.1 - 5.2) | 3.74 (2.64 - 6.68) | 0.79 | 3.6 (3.1 - 5.5) | 3.9 (2.8 - 5.2) | 3.8 (3.1 - 5.1) | 5.3 (2.8 - 7.3) | 0.88 |
| Last 2 years | 4.4 (3.4 - 6.1) | 4.21 (2.73 - 6.24) | 0.32 | 4.4 (3.3 - 6.5) | 4.3 (3.1 - 5.9) | 3.7 (2.8 - 5) | 5.2 (4 - 7.6) | 0.56 |
|  | | | | | | | | |
| **Participants with HbSS (N=99)** | | | | | | | | |
|  | **Sex** | | | **Socio-economic status*** (Statewide median income=$63575) | | | | |
|  | **Male** | **Female** | ***P* value** | **Group 1** | **Group 2** | **Group 3** | **Group 4** | ***P* value** |
| **HbF**, median (IQR) | | | | | | | | |
| Most recent | 15.8 (9-27) | 14.9 (8.9-29.4) | 0.99 | 15.5 (6.6-27.6) | 14.5 (8.9-30.3) | 16 (7.5-26) | 19 (11.8-27) | 0.73 |
| Last year | 17.9 (10.9-28.4) | 18.6 (8.7-29.4) | 0.94 | 18.8 (10.3-26) | 17.2 (9.8-28.1) | 25.6 (7-34) | 18 (13.9-24.5) | 0.78 |
| Last 2 years | 16.5 (10.5-24.7) | 18.1 (12.4-25.6) | 0.42 | 15.2 (5.1, 25.6) | 17.1 (10.8-24.4) | 22.6 (7.9-29.7) | 16.8 (15-19) | 0.68 |
| **MCV**, median (IQR) | | | | | | | | |
| Most recent | 101.7 (94.5-109) | 102 (91.7-113) | 0.93 | 99.6 (92.6-107.7) | 100 (91.7-112.6) | 105.8 (97.5-112.7) | 108 (94.8-119.8) | 0.29 |
| Last year | 105.8 (96-114.6) | 95.6 (91-107.6) | **0.02** | 98.5 (95-108.8) | 99 (90-114) | 100 (94-115.6) | 104.5 (95.5-119.8) | 0.71 |
| Last 2 years | 101 (96-112) | 98.5 (92.4-112.9) | 0.51 | 98.6 (94.8-104.9) | 100 (91.6-112.9) | 106 (98-115.9) | 101 (96.9-112.9) | 0.48 |
| **ANC**, median (IQR) | | | | | | | | |
| Most recent | 3.8 (2.3-6.5) | 3.8 (2.7-5.7) | 0.98 | 4.5 (3-5.9) | 4 (2.4-6.5) | 3.5 (2.5-5.4) | 3 (2-6.4) | 0.72 |
| Last year | 3.9 (3-5) | 3.7 (2.7-6.6) | 0.91 | 3.6 (3.1-5.5) | 3.9 (2.8-5.9) | 3.4 (3-5) | 4.9 (2.8-7.3) | 0.79 |
| Last 2 years | 4 (3.5-6) | 4.3 (2.8-6.2) | 0.46 | 4.4 (3.3-6.5) | 4.3 (3.2-6.1) | 3.6 (2.8-4.6) | 4.8 (4-7.6) | 0.44 |

ANC: absolute neutrophil count; HbF: fetal hemoglobin; MCV: mean corpuscular volume

Group 1: <60% of statewide median (<$38145), Group 2: 60-80% of state-wide median ($38145-$63575), Group 3: >100% to 140% of statewide median ($63575 to $89005), Group 4: >140% of state-wide median (>89005)

P value <0.05 was statistically significant
